# Attitudes Toward AI Usage in Patient Health Care: Evidence From a Population Survey Vignette Experiment

**DOI:** 10.2196/70179

**Published:** 2025-05-27

**Authors:** Simon Kühne, Jannes Jacobsen, Nicolas Legewie, Jörg Dollmann

**Affiliations:** 1 Faculty of Sociology Bielefeld University Bielefeld Germany; 2 Data-Method-Monitoring Cluster German Center for Integration and Migration Research Berlin Germany; 3 Institute of Sociology University of Münster Münster Germany; 4 Mannheim Centre for European Social Research (MZES) University of Mannheim Mannheim Germany

**Keywords:** artificial intelligence, trust, public attitudes, patient health care, survey research, vignette experiment

## Abstract

**Background:**

The integration of artificial intelligence (AI) holds substantial potential to alter diagnostics and treatment in health care settings. However, public attitudes toward AI, including trust and risk perception, are key to its ethical and effective adoption. Despite growing interest, empirical research on the factors shaping public support for AI in health care (particularly in large-scale, representative contexts) remains limited.

**Objective:**

This study aimed to investigate public attitudes toward AI in patient health care, focusing on how AI attributes (autonomy, costs, reliability, and transparency) shape perceptions of support, risk, and personalized care. In addition, it examines the moderating role of sociodemographic characteristics (gender, age, educational level, migration background, and subjective health status) in these evaluations. Our study offers novel insights into the relative importance of AI system characteristics for public attitudes and acceptance.

**Methods:**

We conducted a factorial vignette experiment with a probability-based survey of 3030 participants from Germany’s general population. Respondents were presented with hypothetical scenarios involving AI applications in diagnosis and treatment in a hospital setting. Linear regression models assessed the relative influence of AI attributes on the dependent variables (support, risk perception, and personalized care), with additional subgroup analyses to explore heterogeneity by sociodemographic characteristics.

**Results:**

Mean values between 4.2 and 4.4 on a 1-7 scale indicate a generally neutral to slightly negative stance toward AI integration in terms of general support, risk perception, and personalized care expectations, with responses spanning the full scale from strong support to strong opposition. Among the 4 dimensions, reliability emerges as the most influential factor (percentage of explained variance [EV] of up to 10.5%). Respondents expect AI to not only prevent errors but also exceed current reliability standards while strongly disapproving of nontraceable systems (transparency is another important factor, percentage of EV of up to 4%). Costs and autonomy play a comparatively minor role (percentage of EVs of up to 1.5% and 1.3%), with preferences favoring collaborative AI systems over autonomous ones, and higher costs generally leading to rejection. Heterogeneity analysis reveals limited sociodemographic differences, with education and migration background influencing attitudes toward transparency and autonomy, and gender differences primarily affecting cost-related perceptions. Overall, attitudes do not substantially differ between AI applications in diagnosis versus treatment.

**Conclusions:**

Our study fills a critical research gap by identifying the key factors that shape public trust and acceptance of AI in health care, particularly reliability, transparency, and patient-centered approaches. Our findings provide evidence-based recommendations for policy makers, health care providers, and AI developers to enhance trust and accountability, key concerns often overlooked in system development and real-world applications. The study highlights the need for targeted policy and educational initiatives to support the responsible integration of AI in patient care.

## Introduction

### Background

Ongoing developments in artificial intelligence (AI) accelerate the hopes and potential for improving patient care, diagnostics, and therapy at scale. For years, AI tools in medical imaging and image-based diagnostics have been used extensively in disease detection (eg, tuberculosis from chest radiographs or malignant melanoma from skin images [1]). Applications include analysis of a variety of image technologies such as X-rays, computed tomography, magnetic resonance imaging, and microscopy using machine learning techniques ranging from supervised and unsupervised learning to deep learning [2,3]. Compared with human (radiology) assessment of images, AI tools have regularly shown superior results [4]. Other successful examples of AI usage in medical contexts include drug development, surgery, and medical management ([5] and [6] for an overview).

More recently, generative AI and large language model–based chatbots such as Open AI’s ChatGPT received substantial attention and have promising uses in the health sector [7]. Existing applications include symptom checkers [8], treatment recommendations [9], and diagnostic pathology [10]. Potentially, (generative) AI systems may be used even more broadly for diagnosis and treatment, thereby lowering the burden on medical staff, or even replacing parts of the personnel in the long term and taking over tasks such as communication with patients, managing patient health data, diagnoses, and treatment planning.

Integrating AI technology into health care at scale requires general trust toward and acceptance of AI use in medical contexts. Consequently, a deeper understanding of potential concerns is necessary for an ethical integration of AI into health care. Thus far, empirical evidence on the factors of public trust toward AI in patient health care is limited.

This article addresses this gap by applying a multidimensional, factorial (vignette) design to study the relative and combined importance of AI attributes for public support when implemented in fictional patient health care settings. Based on a probability sample of more than 3000 respondents from Germany’s general population, we study the effects on general support, risk perception, and perception of personalized care of AI. In addition, we investigate the relevance of patient characteristics and analyze potential heterogeneous effects. Our results represent unique insights into the factors that potentially drive the acceptance of AI system integration into medical diagnosis and treatment across various sociodemographic population groups. In the following, we provide an overview of the theoretical background and empirical evidence on attitudes toward AI as well as applications to public health research, before specifying our research questions and study contributions.

### Attitudes and Trust Toward AI

While the technological potential appears vast, numerous issues, risks, and challenges arise in the application of AI in health-related contexts. These stem from both the unique characteristics of AI and the level of public acceptance. On the AI system side, research has identified bias as a potentially critical issue for implementation. For instance, existing research has highlighted how systemic biases and inequalities regarding race and gender can shape the design, implementation, and use of AI technologies, with biased data sets, misapplication, and misplaced trust in AI fairness being major factors in this translation process [11-17]. Furthermore, legal aspects come into play when AI is applied to the health sector with open questions regarding responsibility [18], liability [19], and data protection [20].

Public attitudes toward AI are crucial in these contentious processes. They can influence the opinions of elected representatives, lend legitimacy to design choices or regulatory efforts, shape public awareness and user behavior with AI technologies, and affect the design, rollout, and monitoring of new technologies. In a systematic review of 23 studies addressing attitudes toward clinical AI, Young et al [21] conclude that “patients and the general public conveyed positive attitudes toward AI but had many reservations and preferred human supervision.”

Attitudes toward AI technologies hinge on the attributes of patients on the one hand and the characteristics of the AI system on the other hand [22]. On the patient side, several sociostructural and psychological factors have been identified that shape attitudes toward technology and AI. Positive attitudes toward AI are more likely in people with more technology or computer experience, knowledge about AI, certain personality traits (eg, agreeableness), and younger, female, and higher educated persons [23-26]. Furthermore, trust among migrant populations is an important dimension in health care studies and beyond [27,28]. On the AI system side, no universal and widely accepted set of properties exists in the literature. Rather, existing studies and attempts at a framework (or theory) propose different sets of dimensions and AI properties. For example, De Freitas et al [22] identify 5 main sources of resistance against AI: opacity (AI as a black box), emotionlessness (AI as unfeeling), rigidity (AI as inflexible), autonomy (AI as in control), and group membership (AI as nonhuman). Glikson and Woolley [29] summarize key dimensions for trust in AI, including tangibility (physical and visual), transparency, reliability, task characteristics (eg, technical vs social), and immediacy behaviors (eg, responsiveness and personalization). Thiebes et al [30] list beneficence, nonmaleficence, autonomy, justice, and explicability as key dimensions for trust in AI. Finally, Kaur et al [31] propose fairness, explainability, accountability, privacy, and acceptance as key dimensions for trustworthy AI. While these contributions offer insights into potential dimensions of trust toward AI, they do not represent tested and widespread theoretical frameworks. In this regard, many studies refer to 2 key frameworks of technology acceptance and usage underlying attitudes toward technology (including, in principle, AI): the Technology Acceptance Model (TAM, Davis [32]), highlighting perceived usefulness and perceived ease of use, and the Unified Theory of Acceptance and Use of Technology (UTAUT, Venkatesh et al [33]), including four key factors: performance expectancy, effort expectancy, social influence, and facilitating conditions.

With respect to measurement in survey contexts, several scales have been developed that aim at measuring attitudes toward AI. Sindermann et al [34] propose the Attitude Toward Artificial Intelligence (ATAI) scale, including 5 items on fear, trust, threats and advantages for humanity, and unemployment. The General Attitudes towards Artificial Intelligence Scale (GAAIS) was proposed by Schepman and Rodway [35] and covers dimensions such as opportunities, benefits, positive and negative emotions, and concerns. Stein et al [36] propose the ATTARI-12 (Attitudes toward Artificial Intelligence) scale for measuring attitudes toward AI, focusing on the cognitive, affective, and behavioral facets of attitudes and their relationship with personality traits. Finally, Gnambs et al [37] apply the ATTARI-12 scale to work, health care, and educational domains and develop the ATTARI in Work, Health care, and Education (ATTARI-WHE) scale, which measures 3 distinct factors (cognitive, affective, and behavioral) and shows variation across domains with less positive attitudes for health care applications.

In summary, trust toward AI in medical contexts and patient health care depends on several factors across multiple dimensions. On the patients’ side, personal experiences, sociodemographic background, and personality traits can systematically influence attitudes toward AI. Related to the properties of an AI system, many potential dimensions are mentioned in the literature, and no universal framework exists.

With respect to the (fictional) use case adapted in this article, representing a current generation, nontangible AI system that relies on software and computation not directly visible to patients, we identified key dimensions to be tested for their relative importance of shaping attitudes toward AI in a medical context: autonomy, costs, reliability, and transparency. Autonomy relates to the extent of independence in decisions (and consequences) and the degree to which humans can collaborate and intervene. Costs relate to the efficiency of AI systems compared with existing solutions and non-AI procedures. Reliability includes the usefulness and accuracy of AI systems compared with humans. Transparency relates to whether solutions developed by the AI system are transparent and comprehensible for humans.

The selection of the 4 AI dimensions was guided by 3 criteria. First, these dimensions are particularly relevant for our use case of AI in public (hospital) health care and reflect core concerns in current debates, namely, the degree to which AI systems operate independently from human oversight (autonomy and transparency) and societal expectations around their effectiveness (reliability) and efficiency (costs). Second, they are used frequently in empirical studies and conceptual frameworks on public perceptions of AI and AI design ([22,29-31]). Third, the selected dimensions are based on established models of technology acceptance and use, in particular the TAM and the UTAUT. These frameworks highlight key factors that affect attitudes toward new technologies (perceived usefulness, ease of use, and facilitating conditions), which can be related to the dimensions examined in our study. In particular, reliability reflects expectations of improved performance (usefulness), transparency relates to the ease of understanding and interacting with the system (ease of use), autonomy addresses the degree to which users maintain control and supervision (ease of use), and costs related to whether users perceive sufficient infrastructure or resources to support AI use (facilitating conditions). While other dimensions, such as privacy or fairness, are certainly important in broader discussions of AI ethics, the 4 selected dimensions are particularly well-suited in the context of health care and AI applications aimed at the public. They are also conceptually distinct and complementary, which makes them appropriate for inclusion in a compact, multi-item (factorial) survey experiment.

Applying these dimensions, we shed light on 2 health care areas, diagnosis and treatment, and analyze attitudes toward AI implementation in these application settings separately.

Related to the outcome side (attitudes toward AI), several dimensions have been studied, including trust, acceptability or general support, and perceived risks (Young et al [21] for a systematic review). For the given study, we focus on general support and perceived risks. In addition, we measured individuals’ perception of receiving personalized care, meaning treatment or diagnosis that is adjusted to their individual needs.

### Acceptance of AI in Patient Health Care

Several studies investigate the perception of and attitudes toward AI in health care, including topics such as perceived benefits, risks, or fairness. A qualitative study by Gould et al [38] explored the understanding and attitudes of 20 knee replacement surgery patients regarding AI in risk perception for shared clinical decision-making. The study identified 3 main themes: expectations, empowerment, and partnership, highlighting patients’ desire for self-determination, the potential of AI to provide personalized risk information, and the need for a symbiotic relationship between AI and clinicians. Mikkelsen et al [39] investigated patient perspectives on trust in the patient-general practitioners (GP) relationship, data sharing, and AI use in general practice through qualitative interviews with 10 patients in the North Denmark Region. Participants expressed high trust in their GPs and were willing to share health data if AI was used as a support tool rather than replacing GPs. The findings suggest that the patients are positive about AI in general practice, provided it maintains the central role of the GP in decision-making. Tran et al [40] document chronic patients’ perceptions of biometric monitoring devices and AI in health care based on a survey of 1183 respondents, revealing that 50% view these technologies as beneficial, while 11% see them as dangerous. Patients expressed concerns about technology replacing human care and preferred AI to assist rather than replace human clinicians, particularly in sensitive or long-term care situations. Yap et al [41] surveyed 438 diabetic retinal screening patients in New Zealand to evaluate their perspectives on AI use. Despite 73% being aware of AI and 78% being comfortable with its use, only 58% knew of its implementation in health care, and trust varied, with younger participants showing less trust in AI systems. Most participants preferred clinician involvement in screening, with the perceived benefits of AI being faster and more accurate diagnostics.

While survey studies that analyze AI attitudes based on a fixed set of items offer valuable insights into the perceived importance of isolated characteristics of AI systems, they often do not enable in-depth analysis of the relative importance of characteristics across potential scenarios. In this regard, survey-experimental techniques such as factorial designs (vignette analysis) or conjoint analysis allow for analyzing multidimensional preferences and estimating (causal) effects of multiple attributes simultaneously [42,43]. Compared with standard survey questions and items, factorial survey experiments offer several advantages including (1) ease in comparing the relative influence of different factors on outcomes, (2) higher contextual realism as scenarios (vignettes) mimic real-world complexity often leading to more ecologically valid findings, and (3) improved causal inference due to random assignment in an experimental setting.

Thus far, and to the best of our knowledge, only 2 studies have used survey-experimental designs to investigate multidimensional attributes of medical AI systems and their (public) perception. Ploug et al [44] conducted “a choice-based conjoint survey of public preferences for attributes of AI decision making in health care in a representative sample of the adult Danish population”. The authors compare 6 attributes of AI systems that were selected based on focus group interviews, including accuracy, possibility of discrimination, or responsibility for the final decision. The authors analyze the effects of these attributes on the choice of one out of 3 hypothetical hospitals with AI system integration. The results indicate that whether “physicians are ultimately responsible for diagnostics and treatment planning” is the most important factor for hospital choice, followed by the explainability of the system and whether the system was tested for discrimination. While their results are clearly informative, we see several limitations. First, the study is based on a rather small nonprobability sample of about 1000 participants, thereby limiting generalizability and statistical power with respect to subgroup analyses. Second, since system preference is the only outcome under study, the authors present no insights into perceived usefulness or risks. Finally, the relevance of AI properties in diagnostics versus treatment cannot be fully separated based on the applied research design.

Nurek and Kostopoulou [45] conducted a vignette-based experiment based on 730 UK adults. They investigated the role of doctors’ use of AI-based diagnostic decision aids (DDA) for patients’ evaluation of doctors and found a positive effect of DDA usage on satisfaction and likelihood of recommendation of doctors. Again, the study is clearly informative and well-designed but leaves room for further investigation. First, participants were recruited from a nonprobability online access panel, thereby limiting the generalizability of results. Second, the DDA system mentioned in the vignettes was always introduced as a “differential diagnosis generator, providing the physician with a list of possible diagnoses to consider, and recommending a specific test to rule out a serious disease” [45] with no variation in system properties. Consequently, varying AI system characteristics cannot be compared with each other. Finally, the main study outcomes relate to doctor recommendations only and do not include attitudes toward AI.

### Research Questions and Study Contributions

This article investigates two overall research questions:

1. How important are the properties of an AI system (autonomy, costs, reliability, and transparency) in hypothetical health care settings (diagnosis and treatment) for public attitudes (support, risk, and personalized care)?
2. What role do individual sociodemographic characteristics play in the relationship between AI properties and these outcomes?

Given the limited empirical evidence on the topic, this article contributes to the literature in several ways. It is, to the best of our knowledge, the first empirical contribution to AI perception and acceptance for diagnosis and treatment that (1) is based on a large-scale probability sample of a country’s general population, (2) uses a factorial survey (vignette) experiment to study the relative and multidimensional importance of AI attributes for their perceived usefulness and risks in the public, and (3) models potential heterogeneous effects of AI system properties on those outcomes across several sociodemographic groups.

Our study focuses on Germany. Public attitudes toward AI in health care are shaped by national contexts, including institutional structures and regulatory cultures [46]. The German health care system is based on mandatory insurance for all citizens, ensuring nearly universal access to medical care. It is financed through income-dependent contributions jointly paid by employees and employers. Statutory health insurance, which covers most of the population, operates under the principles of solidarity, where healthy and affluent members contribute to the financing of care for the sick and economically disadvantaged. The mandatory insurance and solidarity principle mean that no one can evade substantial reforms or their funding. In Germany, it is therefore essential to assess whether substantial changes, such as the use of AI in diagnostics and treatment, receive public support to safeguard trust in the health care system and its legitimacy. The German system is also characterized by a close doctor-patient relationship, with general practitioners playing a central role. Compared with more market-oriented systems, such as those in the United States, Germany’s health system characteristics may lead to distinct public attitudes. Furthermore, Germany’s cautious approach to digital technologies and strong data protection norms contribute to more skepticism toward technology, particularly in sensitive areas such as health [47,48]. Against this backdrop, it is crucial to examine how people in Germany perceive AI-based health care interventions.

## Methods

### Sample and Survey

We use data from the DeZIM.panel, a general population survey of people living in Germany who are 18 years or older [49]. Topics include attitudes, income, health, values, and discrimination experiences. The DeZIM panel is an ongoing panel study that started in 2021 and is carried out by the German Centre for Integration and Migration Research (DeZIM). Each year, 4 short waves are carried out. The sampling of respondents was stratified by country of origin, resulting in an oversampling of the most important immigrant cohorts in Germany: former guest workers, late expatriates from the former Soviet Union, immigrants from Muslim countries, and Turkey.

We worked with the ninth wave (December 2023 to February 2024) of the DeZIM.panel (N=3674). Since some respondents did not answer all questions after the vignette, we reduced the sample to those respondents who answered all relevant questions (N=3030) so that coefficients could be compared across models. All data is available to the scientific community through the DeZIM research data center (DeZIM.fdz [50]).

Due to the oversampling of immigrants, people who immigrated to Germany or their children are overrepresented in the sample. This also affects the slightly different age and gender distribution in the sample. Since we are using an experimental and counterfactual design, our results can nevertheless still be interpreted in a causal manner [51], and we refrained from using survey weights for estimation as our focus is on relationships rather than population point estimates, and weights would lead to less efficient estimates.

### Measurement and Vignette Experiment

We used a multidimensional, factorial (vignette) design to study the importance of AI attributes (implemented in patient health care) on general support, risk perception, and personalized care expectation. The introduction to our vignette described a fictional scenario in hospital health care (an example scenario can be found in Section A1 in Multimedia Appendix 1):

In the following, we describe two different cases in which artificial intelligence (AI) could be used in the healthcare sector. We will then ask you a few questions.

In a municipal hospital, artificial intelligence is used to relieve the doctors...

Each respondent was then presented with two vignettes, one relating to diagnosis and one related to treatment planning (therapy). The presentation of the two vignettes was randomized among respondents to counteract order effects; some received the vignette for “diagnosis” first, while others received the “treatment” vignette first. Based on the aforementioned theoretical and empirical frameworks, we applied 4 dimensions to our vignette experiment. Each dimension included a set of textual variations (Textbox 1).

**Textual variations across vignette dimensions.**

**Autonomy: extent of artificial intelligence (AI) system implementing decisions.**

- “...The AI collaborates with doctors. Doctors make the decisions and communicate them to the patients...”
- “...The AI makes decisions independently and communicates them to patients...”

**Costs: cost efficiency of the AI system.**

- “...Through the use of AI the costs of diagnosis/treatment will halve over a period of 10 years...”
- “...Through the use of AI the costs of diagnosis/treatment remain roughly the same over a period of 10 years...”
- “...Through the use of AI the costs for diagnosis/treatment double over a period of 10 years...”

**Reliability: accuracy of the system compared with humans**

- “...Compared with doctors, AI makes wrong decisions more often...”
- “...Compared with doctors, AI makes wrong decisions less often...”
- “...Compared with doctors, AI makes wrong decisions just as often...”

**Transparency: extent of decisions made by the AI system being transparent and comprehensible for humans**

- “...The AI is traceable for doctors and is constantly monitored.”
- “...The AI is partially traceable for doctors and is monitored from time to time.”
- “...The AI is not traceable for doctors and is not monitored.”

For each dimension, a text variation was assigned randomly to all respondents. The total number of vignettes amounts to 2 × 3 × 3 × 3 = 54 scenarios. On average, each scenario was presented to 56 (SD 6.1; minimum 45, maximum 70) respondents.

A series of follow-up questions was presented to respondents after each of the two vignettes (diagnosis and treatment).

1. General support: “How much would you be in favor of or against the use of the AI described? (1 (I am fully in favor)...7 (I reject this completely))”
2. Risk perception: “How safe or risky do you think the use of the AI described is for patients?(1 (very safe)...7 (very risky))”
3. Personalized care: “Imagine that you have to go to the hospital described above with an illness that requires the use of AI. On a scale of 1 to 7, how likely do you think it is that you will receive a diagnosis/treatment that is personalized to you and your personal characteristics? (1 (very likely)...7 (very unlikely))”

### Identification Strategy and Statistical Analysis

To test the relative influence of each vignette component on the dependent variables, we used linear regression analyses with robust SEs and all dimensions in the model. Since respondents only saw one vignette for each application area (diagnosis and treatment), multilevel models were not necessary. Due to the random display of components within dimensions, we did not use any other covariates or weighting in the model. For the interpretation of results, we show the unstandardized regression coefficients b, displaying the mean differences between components within each dimension. We estimated separate models for all combinations of outcomes (general support, risk perception, and personalized care) and scenarios (diagnosis and treatment), resulting in 6 models. We discuss and compare models for diagnosis and treatment separately.

Besides the relative influence of each component in a dimension, we estimated how much variance of the dependent variable can be explained by each dimension. This allows for a convenient overview of the relative importance of AI properties on the outcomes.

Finally, we studied potential heterogeneous effects across sociodemographic patient dimensions by estimating separate regression models and comparing effect estimates across population subgroups (gender, age, educational level, migration background, and subjective health status compared with the average), again separately for diagnosis and treatment. Subgroup effect estimates are inherently more uncertain than main effects due to smaller subsample sizes. In this context, statistical power (the ability to detect a true effect) is a crucial aspect. Since our vignette experiment was embedded in an ongoing panel survey, we had no control over subsample sizes to conduct a priori power calculations. However, we can estimate the required subgroup sample sizes based on the following assumptions: a 2-tailed test with a significance level of α=.05, 80% statistical power, approximately equal subgroup sizes, and an average SD of about 1.8. Under these conditions, the necessary sample size per subgroup was 204 for an effect size of 0.5 and 1272 for an effect size of 0.2. This range aligns with many of the subgroup sizes. While we consider our study sufficiently powered to analyze overall patterns related to heterogeneous effects, it may nonetheless be slightly underpowered to detect smaller effects among subgroups.

### Ethics Approval

Ethical approval for this study was granted by the Ethics Committee of the German Center for Integration and Migration Research (DeZIM, case EK-03/2024). All respondents provided written consent before participating in the survey. Participation was voluntary, and a written explanation of data security, privacy, and storage was presented before participation. Compensation for participation in the DeZIM.panel is €10 (US $11.36) per survey year.

## Results

Table 1 summarizes the unweighted sociodemographic characteristics of the analytical sample.

**Table 1:** Sociodemographic sample characteristics.

Characteristics			Values, n (%)
**Gender**			
	Men	1471 (48.6)	
	Women	1554 (51.3)	
	Missing	5 (0.2)	
**Age groups (years)**			
	18-27	489 (16.1)	
	28-37	789 (26.0)	
	38-47	553 (18.3)	
	48-57	595 (19.6)	
	58-68	594 (19.6)	
	Missing	10 (0.3)	
**Education (condensed ISCED^a^)**			
	Primary or none	225 (7.4)	
	Secondary	1094 (36.1)	
	Tertiary	1677 (55.4)	
	Missing	34 (1.1)	
**Migration background**			
	None	1990 (65.7)	
	Immigrant	584 (19.3)	
	Direct descendant of an immigrant	437 (14.4)	
	Missing	19 (0.6)	
**Health compared with average**			
	Better	965 (31.9)	
	About the same	1432 (47.3)	
	Worse	618 (20.4)	
	Missing	15 (0.5)	

^a^ISCED: International Standard Classification of Education.

### General Support, Risk Perception, and Personalized Care

Table 2 shows the mean, SD, median, minimum, and maximum of all dependent variables. Averages ranging from 4.2 (SD 1.7) to 4.4 (SD 1.8) on a 1- to 7-point scale (and larger numbers represent less support, more risks, and fewer expectations for personalized care) suggest a generally neutral or slightly unsupportive learning overall. The full range of the scale was used, showing variability in participant opinions from strong support to strong opposition. The little variation in means between either diagnosis and treatment or between general support, risk perception, or personalized care indicates only limited differentiation by respondents across outcomes and settings.

**Table 2:** Descriptives of dependent variables (N=3030).

Variable			Mean (SD)		Median (IQR)		Minimum/maximum			
**Diagnosis**										
	General support			4.3 (1.9)		4 (3-6)		1/7		
	Risk perception		4.4 (1.7)		4 (3-6)		1/7			
	Personalized care		4.2 (1.7)		4 (3-6)		1/7			
**Treatment**										
		General support	4.4 (1.8)		4 (3-6)		1/7			
		Risk perception	4.4 (1.7)		4 (3-6)		1/7			
		Personalized care	4.2 (1.7)		4 (3-6)		1/7			

### Attitudes Toward AI in Diagnosis

**Figure 1:**
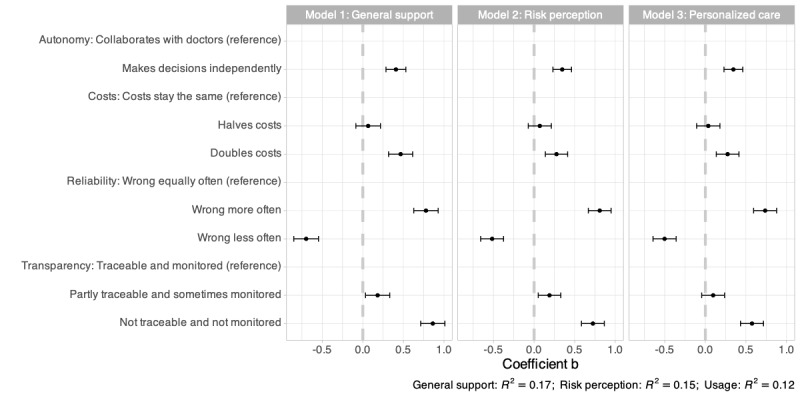
Linear regression results for general support (model 1), risk perception (model 2), and personalized care (model 3) related to diagnosis. Positive coefficients (effect sizes) represent lower general support, higher perceived risks, and lower beliefs in personal, customized services and care.

Figure 1 presents the unstandardized effect sizes (b) for 3 models examining effects on general support, risk perception, and personalized care in relation to diagnosis. A table displaying the coefficients can be found in Table S1 in Multimedia Appendix 1. Positive coefficients (effect sizes) represent lower general support, higher perceived risks, and lower beliefs in personal, customized services and care.

#### General Support

Based on the results, several dimensions show notable effects on the level of support of AI usage in diagnosis. In the autonomy dimension, AI making decisions independently is associated with a significant effect (b=0.41, *P*<.001) compared to AI working with doctors, and thus, signaling a lower support of AI when it works independently. In the dimension of the cost, halving costs shows merely an effect compared with the reference category where costs remain the same (b=0.07, *P*=.39). However, doubling the costs is linked to a substantial effect (b=0.47, *P*<.001), implying that higher costs are strongly associated with lower support of AI in diagnosis. Related to reliability, we observe a highly significant effect (b=0.78, *P*<.001) of AI being wrong more often compared with the reference category (wrong equally often), representing lower support of AI. In accordance, AI being wrong less often leads to a reverse effect (b=–0.70, *P*<.001), thus, higher support of AI. Finally, in the transparency dimension, we observe a strong and significant effect of AI being not traceable and not monitored (b=0.86, *P*<.001) compared with the reference category (AI is traceable and monitored). This shows that the transparency of AI in diagnosis is a particularly important factor for support of AI usage. Partially traceable and monitored AI is disapproved only slightly more than fully traceable and monitored AI (b=0.18, *P*=.02).

#### Risk Perception

Related to risk perception of AI usage in diagnosis, the results follow a similar pattern as with the previous model on support. Independent decision-making of AI is evaluated as riskier (b=0.35, *P*<.001), as are higher costs (b=0.28, *P*<.001), making wrong decisions more often (b=0.81, *P*<.001), and AI not being traceable and or monitored (b=0.72, *P*<.001).

#### Personalized Care

Model 3 investigates the effects of AI characteristics on the perceived likelihood of receiving a personalized diagnosis. Again, the pattern strongly resembles the previous results with personalized care perceived as more unlikely in the case of independent decision-making of the AI system (b=0.35, *P*<.001), higher costs (b=0.27, *P*<.001), more erroneous results due to AI (b=0.74, *P*<.001), and AI being not traceable and not monitored (b=0.57, *P*<.001).

### Attitudes Toward AI in Treatment

**Figure 2:**
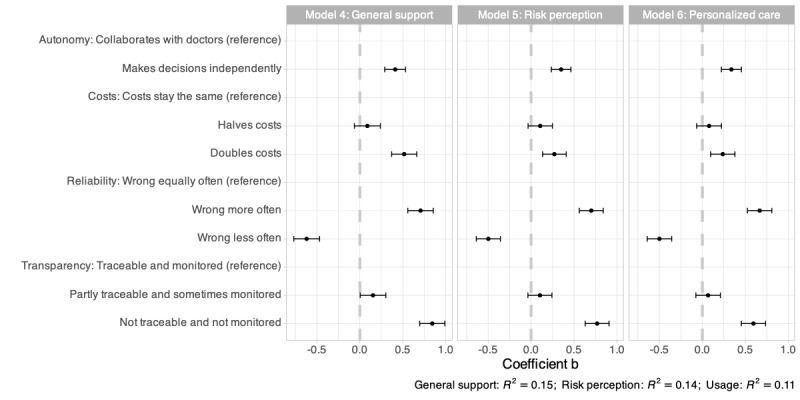
Linear regression results for general support (model 4), risk perception (model 5), and personalized care (model 6) related to treatment. Positive coefficients (effect sizes) represent lower general support, higher perceived risks, and lower beliefs in personal, customized services and care.

Figure 2 presents the unstandardized effect sizes (b) for 3 models examining levels of support, risk perception, and personalization in relation to treatment. A table displaying the results can be found in Table S2 in Multimedia Appendix 1. Again, positive coefficients (effect sizes) represent lower support, higher perceived risks, and lower beliefs in personal, customized services.

Generally, the results represent a highly similar pattern compared to models 1-3 on diagnosis. Significant effects on all 3 outcomes (general support, risk perception, and personalized care) are present for AI making decisions independently, increased costs, being wrong more often (and less often), and not being traceable or monitored.

### Regression Results Summary

In summary, the regression results highlight 2 overall patterns. First, participants do not substantially differentiate between AI usage in diagnosis versus treatment. Comparing the results for diagnosis (Figure 1) and treatment (Figure 2) shows highly similar patterns and effect sizes. Second, participants’ support, perceived risks, and beliefs in personalized services are affected very equally by AI characteristics. In other words, participants evaluate AI as generally better (being more supportive, seeing fewer risks, and belief in more personalization) or generally worse (being less supportive, seeing more risks, and not believing in personalized care) based on varying levels of autonomy, costs, reliability, and transparency. Overall, the results point to the formation of a general attitude toward AI usage in patient health care based on the vignette scenario, without further differentiating between applications (diagnosis vs treatment) and consequences (support vs risks vs personalized care).

### Explanatory Power Across Dimensions

**Figure 3:**
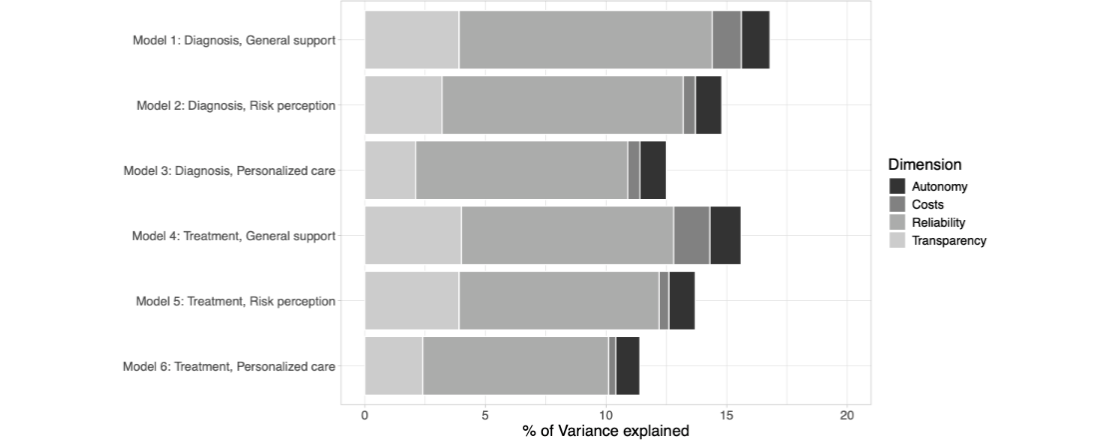
Explained variance across vignette dimensions and outcomes.

Figure 3 displays the cumulative explained variance across the 6 models and for each vignette dimension (Table S3 in Multimedia Appendix 1 for a table with exact numbers). Overall, the cumulative explained variance ranges between 11.4% (model 6: treatment and personalized care) and 16.8% (model 1: diagnosis and support). The most important factor explaining response variation in the outcomes is the reliability of the AI system, accounting for the majority of explained variance in all 6 models, ranging from 7.8% in model 6 (treatment and personalized care) to 10.5% in model 1 (diagnosis and support). Transparency of the AI system (ie, traceable and monitored) is another comparatively important factor with values ranging from 2.1% (model 3: diagnosis and personalized care) to 4.0% (model 4: treatment and support). Contrarily, AI system autonomy and costs play a minor role in explaining response variation in our vignette experiment.

Due to collinearity, the cumulative explained variance of individual variables in a regression model does not equal the model’s total explained variance. We still present the results in Figure 3 using an additive approach to enhance readability since, in our case, the deviations between the cumulative and total explained variance are minimal.

### Heterogeneous Effects Across Sociodemographic Characteristics

**Figure 4:**
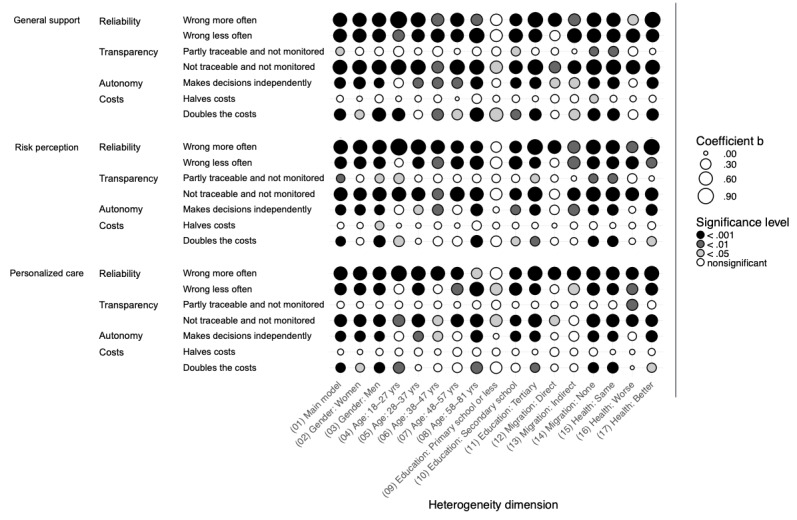
Heterogeneity analysis for diagnosis by sociodemographic groups for support, risk perception, and personalized care in diagnosis.

**Figure 5:**
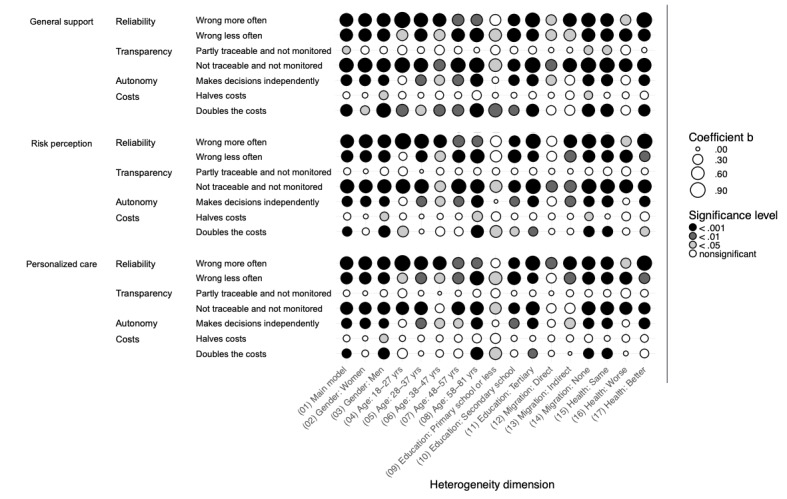
Heterogeneity analysis for treatment by sociodemographic groups for support, risk perception, and personalized care in treatment.

In the following, we examine the potential heterogeneous effects of AI use in diagnosis and treatment on attitudes toward AI use across several sociodemographic groups: gender, age, educational level, migration background, and subjective health compared with the average. Figure 4 (diagnosis) and Figure 5 (treatment) visually present the unstandardized regression coefficients (b) from this heterogeneity analysis based on a total of 102 regression models. Here, dot size reflects effect size, with larger dots representing larger coefficients b, while color denotes significance level, white dots indicate nonsignificant results, and darker dots mark significant results at increasing significance levels. Comparing dots in a column informs about model differences within a sociodemographic group, whereas comparing dots in a row informs about sociodemographic differences within a model. Due to the numerous effects examined, we do not discuss each coefficient individually. A table presenting all effect estimates and SEs can be found in Multimedia Appendix 2 of this article.

In addition, 2 overarching patterns emerge. First, similar to the main models shown in Figures 1 and 2, the differences between diagnosis and treatment outcomes in the heterogeneity analysis are minor. Only on the dimension of costs does a trend emerge, suggesting that various sociodemographic groups perceive AI in treatment settings more critically when associated costs increase, compared with its use in diagnosis. Second, within each dimension, only a few specific effects for particular groups stand out. Notably, individuals with lower education levels show generally smaller and sometimes nonsignificant effects compared with those with higher educational backgrounds (with nonsignificance potentially due to a comparatively low group size of 225).

Gender-based differences are most pronounced in the cost dimension, where men tend to view the increasing costs associated with AI use more critically, particularly in diagnosis. Age-related heterogeneity also appears in the autonomy and cost dimensions, though no clear trend emerges. Instead, coefficient strength and significance fluctuate across age groups without a specific pattern. Furthermore, as mentioned, individuals with lower education levels are less likely to differentiate between various aspects of AI use. Conversely, those with higher education levels critically assess AI applications in the health care system, especially when AI makes autonomous decisions with limited transparency. Individuals without a migration background tend to view AI use more critically in scenarios of low transparency and high AI autonomy in decision-making. Finally, respondents evaluating their health as worse than the average in Germany assess AI applications less critically, specifically in terms of AI making decisions independently and AI being more expensive.

## Discussion

### Principal Findings

The analysis reveals minimal effect heterogeneity across models 1-6, strongly suggesting that respondents hold a general attitude toward AI in patient health care settings without differentiating based on specific application areas such as diagnosis and treatment.

The main findings are:

1. The most pronounced effects are observed in the dimension of reliability: new technology should not introduce any disadvantages in the form of more errors, and the results show that respondents clearly expect new technology to be more reliable.
2. Regarding costs, the relative effect sizes are comparatively small and align with expectations. Higher costs result in rejection, and lower costs garner slight approval. Similar effect sizes are found for autonomy.
3. Here, respondents favor AI systems that collaborate with practitioners and do not make decisions independently.
4. Finally, transparency is of importance to respondents who signal clear disapproval of AI not being traceable and monitored.

The dimensions tested vary in their importance (explained variance). Reliability consistently explains the largest portion of the variance in the dependent variable across all models. Transparency also plays a notable role in all models, while the other two dimensions are almost rendered negligible.

### Analysis Across Sociodemographic Groups

Heterogeneity analysis of AI use in diagnosis and treatment across sociodemographic groups (gender, age, education level, migration background, and subjective health) reveals minimal differences between diagnosis and treatment attitudes. Furthermore, two patterns arise: First, only certain sociodemographic groups exhibit significant effects, with individuals of lower education levels generally displaying smaller and less significant effects than those with higher education. Second, gender differences appear primarily in the cost dimension, where men are more critical of AI use as costs increase, particularly in diagnostic contexts. Age-based heterogeneity also appears in the autonomy and cost dimensions, although no consistent trend across age groups emerges. Notably, individuals without a migration background express heightened concerns about AI when transparency is low and autonomy is high. Those with higher education levels similarly show greater scrutiny toward autonomous AI systems, particularly when transparency is limited. Finally, individuals who perceive their own health as worse than the national average in Germany tend to view AI applications less critically. This is particularly evident in their attitudes toward AI operating autonomously and the potential for higher costs associated with its use.

### Limitations

This study makes a significant contribution to advancing research on public attitudes toward AI in health care. By using a population survey vignette experiment, this work provides a nuanced understanding of general support, risk perception, and personalized care expectations for AI applications in the health sector. While these insights contribute to the current state of research, the study has limitations that should be addressed in future work.

First, despite using a probability-based sample of the German population, the findings may not fully generalize to populations in other countries and regions or health care settings with varying degrees of AI integration. Future studies could benefit from including diverse cultural and health care context comparisons to enhance the generalizability of results. Second, the vignette design, while effective for capturing controlled responses, may simplify complex real-world scenarios, thus limiting the depth of public attitudes captured in a diverse and evolving health care landscape. Third, the study’s focus on autonomy, cost, reliability, and transparency as core AI attributes may overlook other factors that also shape public perceptions. Attributes such as data privacy, personalization needs, and the impact of AI on patient-clinician interactions were not explored in depth. Including a broader set of AI attributes could provide a more comprehensive picture of the factors influencing public acceptance and trust in AI systems for health care applications. Fourth, while this research provides a valuable snapshot of attitudes toward AI in health care at a single time point, it lacks longitudinal data to track how these attitudes might evolve as AI technology and public familiarity continue to advance. Longitudinal studies would be instrumental in understanding shifts in public attitudes, especially as AI applications become more integrated into routine health care practices and as regulatory and ethical frameworks evolve. Fifth, future research could also examine potential interactions between the AI dimensions explored in this study to better understand how multiple characteristics jointly influence attitudes. Identifying whether certain combinations produce particularly strong or weak effects could offer deeper insights into underlying mechanisms. Extending this analysis to 3-way interactions with presumably relevant individual (sociodemographic) factors, including health literacy, health care compliance, and trust in health care institutions, would require sufficiently large and diverse samples to maintain adequate statistical power for detecting complex patterns. Furthermore, future research should consider contextual factors such as national and regional media discourses related to AI and AI in health care, which are likely to shape public attitudes. Such discourses may interact with both individual characteristics and AI attributes, offering important context for interpreting public perceptions.

### Conclusions

Based on a factorial (vignette) experiment implemented in a probability sample from Germany’s general population, our study represents novel insights into the factors that potentially drive the acceptance of AI system integration into medical diagnosis and treatment while also investigating the role of patient sociodemographic characteristics. Taken together, our results indicate a dilemma in the introduction of AI in the health care system: public attitudes toward AI are largely shaped by its characteristics rather than its specific applications. This underscores the need for educational efforts to foster more nuanced opinions, particularly on AI reliability and human oversight, key factors influencing public acceptance. Further research, ideally using mixed methods approaches, is needed to better understand how these attitudes form across different population groups.

## Appendix

Multimedia Appendix 1 Supplementary material.

Multimedia Appendix 2 Coefficients and SEs for the heterogeneity analysis across sociodemographic characteristics.

## Abbreviations

AI: artificial intelligence
ATAI: Attitudes toward Artificial Intelligence
ATTARI-WHE: Attitudes towards Artificial Intelligence in Work, Health care, and Education
DDA: diagnostic decision aid
DeZIM: German Centre for Integration and Migration Research
EV: explained variance
GAAIS: General Attitudes towards Artificial Intelligence Scale
GP: general practitioner
ISCED: International Standard Classification of Education

## Data Availability

The datasets generated or analyzed during this study are available to the scientific community through the DeZIM research data center (DeZIM.fdz [50] for more information). An anonymized dataset and the code (Stata for analysis, R for visualization) for replication are available from the Open Science Framework [52].
