# Supplementary material for: Attitudes Toward AI Usage in Patient Health Care: Evidence From a Population Survey Vignette Experiment
Source: J Med Internet Res. 2025 May 27;27:e70179. doi: 10.2196/70179 (PMC12152429; doi:10.2196/70179)
Supplement: Multimedia Appendix 1 [file jmir_v27i1e70179_app1.docx]

**Supplementary S1: Vignette example text**

“In a municipal hospital, artificial intelligence is used to relieve the doctors. The AI supports the doctors in their work. Doctors make the decisions and communicate them to the patients. Through the use of AI, the costs for diagnosis are halved over a period of 10 years. Compared to doctors, the AI makes fewer incorrect decisions. The AI is transparent for doctors and is continuously monitored.”

**Table S1: Linear Regression Models for Diagnosis**

| Dimension and Variables | | **Model 1: General support** | | **Model 2: Risk perception** | | **Model 3: Personalized care** | |
| --- | --- | --- | --- | --- | --- | --- | --- |
|  |  | Coef.*^1^* | 95% CI | Coef. | 95% CI | Coef. | 95% CI |
| **Autonomy** | |  |  |  |  |  |  |
|  | works with doctors (ref.) |  |  |  |  |  |  |
|  | makes decisions independently | 0.408 | [0.285, 0.530] | 0.347 | [0.232, 0.461] | 0.346 | [0.230, 0.461] |
|  | *P* value | < 0.001 |  | < 0.001 |  | < 0.001 |  |
| **Costs** | |  |  |  |  |  |  |
|  | costs stay the same (ref.) |  |  |  |  |  |  |
|  | halves costs | 0.066 | [-0.085, 0.217] | 0.070 | [-0.072, 0.213] | 0.037 | [-0.106, 0.180] |
|  | *P* value | 0.393 |  | 0.331 |  | 0.614 |  |
|  | doubles costs | 0.466 | [0.318, 0.614] | 0.276 | [0.139, 0.414] | 0.275 | [0.135, 0.414] |
|  | *P* value | < 0.001 |  | < 0.001 |  | < 0.001 |  |
| **Reliability** | |  |  |  |  |  |  |
|  | wrong equally often (ref.) |  |  |  |  |  |  |
|  | wrong more often | 0.778 | [0.628, 0.928] | 0.808 | [0.668, 0.949] | 0.737 | [0.595, 0.879] |
|  | *P* value | < 0.001 |  | < 0.001 |  | < 0.001 |  |
|  | wrong less often | -0.698 | [-0.850, -0.546] | -0.517 | [-0.658, -0.376] | -0.502 | [-0.644, -0.359] |
|  | *P* value | < 0.001 |  | < 0.001 |  | < 0.001 |  |
| **Transparency** | |  |  |  |  |  |  |
|  | traceable and monitored (ref.) |  |  |  |  |  |  |
|  | partly traceable and sometimes monitored | 0.183 | [0.033, 0.332] | 0.190 | [0.051, 0.329] | 0.096 | [-0.044, 0.237] |
|  | *P* value | 0.017 |  | 0.007 |  | 0.180 |  |
|  | not traceable and not monitored | 0.862 | [0.713, 1.011] | 0.724 | [0.584, 0.865] | 0.575 | [0.433, 0.716] |
|  | *P* value | < 0.001 |  | < 0.001 |  | < 0.001 |  |

**Table S2: Linear Regression Models for Treatment**

| Dimension and Variables | | **Model 4: General support** | | **Model 5: Risk perception** | | **Model 6: Personalized care** | |
| --- | --- | --- | --- | --- | --- | --- | --- |
|  |  | Coef.*^1^* | 95% CI | Coef. | 95% CI | Coef. | 95% CI |
| **Autonomy** | |  |  |  |  |  |  |
|  | works with doctors (ref.) |  |  |  |  |  |  |
|  | makes decisions independently | 0.411 | [0.290, 0.533] | 0.350 | [0.236, 0.464] | 0.338 | [0.222, 0.455] |
|  | *P* value | < 0.001 |  | < 0.001 |  | < 0.001 |  |
| **Costs** | |  |  |  |  |  |  |
|  | costs stay the same (ref.) |  |  |  |  |  |  |
|  | halves costs | 0.088 | [-0.064, 0.239] | 0.107 | [-0.035, 0.248] | 0.078 | [-0.066, 0.222] |
|  | *P* value | 0.256 |  | 0.140 |  | 0.286 |  |
|  | doubles costs | 0.517 | [0.371, 0.663] | 0.272 | [0.134, 0.409] | 0.238 | [0.097, 0.379] |
|  | *P* value | < 0.001 |  | < 0.001 |  | 0.001 |  |
| **Reliability** | |  |  |  |  |  |  |
|  | wrong equally often (ref.) |  |  |  |  |  |  |
|  | wrong more often | 0.707 | [0.558, 0.855] | 0.700 | [0.561, 0.839] | 0.667 | [0.525, 0.809] |
|  | *P* value | < 0.001 |  | < 0.001 |  | < 0.001 |  |
|  | wrong less often | -0.619 | [-0.769, -0.469] | -0.498 | [-0.638, -0.357] | -0.499 | [-0.642, -0.356] |
|  | *P* value | < 0.001 |  | < 0.001 |  | < 0.001 |  |
| **Transparency** | |  |  |  |  |  |  |
|  | traceable and monitored (ref.) |  |  |  |  |  |  |
|  | partly traceable and sometimes monitored | 0.153 | [0.004, 0.303] | 0.103 | [-0.037, 0.243] | 0.068 | [-0.075, 0.210] |
|  | *P* value | 0.044 |  | 0.149 |  | 0.354 |  |
|  | not traceable and not monitored | 0.843 | [0.697, 0.990] | 0.769 | [0.630, 0.908] | 0.595 | [0.453, 0.736] |
|  | *P* value | < 0.001 |  | < 0.001 |  | < 0.001 |  |

**Table S3: Explained Variance Across Vignette Dimensions and Outcomes**

|  | Dimension | | | |
| --- | --- | --- | --- | --- |
| **Model** | *Autonomy* | *Costs* | *Reliability* | *Transparency* |
| M1 - Diagnosis: Support | .012 | .012 | .105 | .039 |
| M2 - Diagnosis: Risk Perception | .011 | .005 | .100 | .032 |
| M3 - Diagnosis: Personalization | .011 | .005 | .088 | .021 |
| M4 - Treatment: Support | .013 | .015 | .088 | .040 |
| M5 - Treatment: Risk Perception | .011 | .004 | .083 | .039 |
| M6 - Treatment: Personalization | .010 | .003 | .077 | .024 |
